# Centennials, FOMO, and Loneliness: An Investigation of the Impact of Social Networking and Messaging/VoIP Apps Usage During the Initial Stage of the Coronavirus Pandemic

**DOI:** 10.3389/fpsyg.2021.620739

**Published:** 2021-02-09

**Authors:** Elena Fumagalli, Marina Belen Dolmatzian, L. J. Shrum

**Affiliations:** ^1^School of Business, Universidad Torcuato Di Tella, Buenos Aires, Argentina; ^2^Facultad de Ciencias Humanas y de la Conducta, Universidad Favaloro, Buenos Aires, Argentina; ^3^Department of Marketing, HEC Paris, Jouy-en-Josas, France

**Keywords:** COVID-19, loneliness, social network, messaging apps, fear of missing out, centennials

## Abstract

The current COVID-19 pandemic has had obvious, well-documented devastating effects on people's physical health. In this research, we investigate its potential effects on people's mental health. Many people have experienced social isolation, as countries attempt to stem the spread of the disease through confinement and other forms of social distancing. Intuitively, such social isolation may increase feelings of loneliness, and people may take logical steps to reduce their feelings of social isolation and loneliness. One route is through the use of social networking apps (e.g., Facebook, Instagram) and messaging and VoIP apps (e.g., WhatsApp, iMessage). In this research, we investigate the effects of pandemic-induced social isolation on social networking and messaging apps, and potential related effects on loneliness. We surveyed young adults (*N* = 334) who are part of the Centennial cohort (born after 1995) from three different countries (Italy, Argentina, UK) and obtained their screen time usage data over a 4-week period starting from mid-March 2020. This sampling procedure allowed us to obtain data from respondents who were experiencing different degrees of mandated social isolation (lockdowns), which enabled us to determine whether social network and messaging app usage increased as a function of social isolation, and to test potential effects on levels of loneliness. Results showed that only social network usage increased in the initial stage of confinement as a function of lockdown initiation. Additionally, social network app usage was associated with increased feelings of loneliness, and this relation was mediated by fear of missing out (FOMO). In contrast, messaging app usage was associated with decreased feelings of loneliness, and was unrelated to FOMO. These results suggest that technology may be useful for mitigating the impact of loneliness during social isolation but that it is necessary to promote usage of messaging and VoIP apps, rather than social networking apps, because they are directly associated with decreases in loneliness without increasing FOMO.

## Introduction

As COVID-19 rapidly spread, reaching pandemic levels by early March 2020 (www.who.int), many regional and national governments quickly instituted various measures to mitigate the spread within communities. Along with urgings and mandates to social distance, wear masks, and employ good personal hygiene, mandates requiring social isolation, such as school and business closures, and shelter-in-place requirements, or “lockdowns,” were also widely instituted. Although the positive effects of such social isolation on physical health (avoiding the disease) are now quite apparent, we are only beginning to understand its potential negative effects on mental health. Even before the pandemic and its associated forced social isolation, research suggested that feelings of loneliness were on the rise, to the point of reaching what some referred to as epidemic levels (The Harris Poll, 2016; Noack, 2018; Twenge et al., 2019). Moreover, some research also suggests that the increase in loneliness may be particularly acute for young people, who experience greater changes in loneliness over time in their younger years (Beam and Kim, 2020; Fried et al., 2020), which are linked to the social reorientation period of adolescence (Goossens, 2018).

Clearly, increasing social isolation via lockdowns seems likely to exacerbate an already serious problem for both children and adults. However, one particular factor that may potentially mitigate increased loneliness following forced social isolation is the use of digital communication technologies, such as WhatsApp, Skype, and Zoom, as well as the use of social networks, such as Facebook, Instagram, and Twitter. For example, Banskota et al. (2020) suggest that smartphone applications (apps) that facilitate social communication may help older people stay connected with others, potentially lessening the impact of social isolation on feelings of loneliness. Given that younger people are the most frequent users of such technology, the positive effects of social communication technology may be even greater for them.

Despite the intuitive appeal of this reasoning, a growing body of research suggests that just the opposite may be true: the use of social media technology may actually increase feelings of loneliness, and associated feelings, in young people (e.g., depression, anxiety, low self-esteem, etc.; Kalpidou et al., 2011; Brooks, 2015; Vannucci et al., 2017; Sampasa-Kanyinga et al., 2018; Craig et al., 2020). Our research addresses these questions. In particular, we investigate the effects of forced social isolation brought on by the COVID-19 pandemic on feelings of loneliness in young adults, with a specific focus on the interrelations between social networking apps, messaging and VoIP apps, loneliness, and fear of missing out (FOMO). We tested the interrelations in a multi-country sample (Argentina, Italy, UK) in which the countries initiated social isolation lockdowns at different points over a 4-week period, allowing us to assess the effects of the lockdown on social media usage relative to pre-lockdown periods. In addition, we obtained objective measures of smartphone usage time by having participants upload their smartphone's screen time usage reports, thereby minimizing reporting problems common to self-reports.

## Conceptual Background

### Loneliness

Loneliness is defined as an aversive state that arises from the perception that one's social relationships are deficient (Perlman and Peplau, 1981; Russell et al., 1984; Baumeister and Leary, 1995; Hawkley and Cacioppo, 2010). The perception component of the definition is critical, and indicates that loneliness is a *subjective* experience, and is independent of objective isolation. People who are socially isolated may not feel lonely, and people who have many social connections may nevertheless feel lonely. In other words, it is not so much about the quantity of social connections, but their quality.

The distinction may explain why recent findings related to loneliness following COVID-19-related forced isolation have differed for younger and older adults. For example, in one study that assessed loneliness as a function of number of days following an initial lockdown in a general population sample, self-reported loneliness actually decreased in the first 30 days of the lockdown, before increasing quickly after the first 30 days (Zhang et al., 2020). Possibly, for older adults not living alone, the chance to have quality time with family members may have increased social connectedness. However, recent studies of adolescents and young adults find that adolescents and young adults report suffering significant psychological problems during the COVID-19 pandemic, including depression, anxiety, and loneliness (Varga et al., 2016; Chen et al., 2020; Ellis et al., 2020; Liang et al., 2020). Thus, it may be the case that, unlike older adults, adolescents and young adults may still feel a lack of satisfying social connections even when living with family members.

Developmental processes may also make younger people more vulnerable to feelings of loneliness. Adolescence is a period in which younger people are developing self-identities, and are particularly sensitive to social interaction cues and peer interaction (Orben et al., 2020). Teenagers in normal (non-pandemic) times tend to spend more time with their friends and romantic partners than with their families, and are particularly sensitive to peer rejection (Knoll et al., 2015). Moreover, identity development is associated with changes in social behaviors because social goals change (van den Bos, 2013), a process referred to as social reorientation (Nelson et al., 2005). This model suggests that social goals change during development, so that adolescents are more motivated to seek certain social experiences with their peers (Nelson et al., 2016; Magis Weinberg, 2017), and the process of social reorientation is one of the most salient changes during adolescence (Nelson et al., 2005).

A recent review (Goossens, 2018) suggests that feelings of loneliness tend to be prevalent during adolescence, and that this relationship could be explained by the evolutionary theory of loneliness (Hawkley and Cacioppo, 2010) and the processes of social reorientation characteristic of this developmental period. The theory states that loneliness activates two opposing motives: social re-connection and self-preservation. The self-preservation motive causes lonely individuals to be hypervigilant to social threats, which may lead to worry about and even mis-interpretation of social interactions. Neurological studies further support this reasoning (Somerville, 2013). The regions of the brain that that are activated in response to loneliness are ones that are particularly active during adolescence (Vijayakumar et al., 2017). These developmental differences are also related to reactions to social rejection. At very early ages, children read the social keys of rejection and inclusion. However, the affective responses to these experiences are more exaggerated during adolescence due to an increase in the activity of brain regions that are related to the feeling of loneliness and a decrease in the activity in areas that regulate reactions. In other words, different regions of the brain develop at different times (Somerville et al., 2010). Moreover, the regions related to emotions and reactions develop earlier than regions related to behavior control and emotional regulation. This temporary imbalance in brain development could explain the stronger reaction that adolescents might have to the perception of social rejection, making them more vulnerable to perceived loneliness (Goossens, 2018).

In sum, young people appear to be vulnerable to feelings of loneliness, and pandemic lockdowns may exacerbate the situation by limiting social connection. One potential remedy to pandemic-induced social isolation is the use of digital forms of social interaction, such as social networks (e.g., Facebook) and other digital communications (e.g., WhatsApp), and this is true for both older (Banskota et al., 2020) and younger people (Orben et al., 2020). We address this possibility in the following sections.

#### Social Network Apps Usage and Loneliness

For young people in particular, who are voracious consumers of social media, both the number of social contacts and the frequency of social contacts may increase exponentially through social media compared to face-to-face interactions, which may reduce feelings of loneliness and lack of social connection. However, research both pre-pandemic and since the pandemic's inception seems to suggest the opposite. Recent pre-pandemic studies of adolescents (Barry et al., 2017; Twenge et al., 2019) and young adults (Primack et al., 2017) both found positive relations between loneliness and social media usage. Experimentally manipulating (restricting) social network usage produced similar findings. For example, Hunt et al. (2018) experimentally manipulated social network usage for college undergraduates over the course of 3 weeks. They found that participants in the experimental group, which limited their usage of social media (Facebook, Snapchat, Instagram) to 10 min, per platform, per day, reported lower levels of loneliness, compared to the control group which used the social media platforms as they normally would. The reasoning is that even though social media may increase the quantity of social contacts and interactions, the quality of contacts and interactions may actually decrease. That is, the lower-quality social media interactions may replace or crowd-out more high-quality in-person interactions.

Research since the pandemic's inception also finds that social media use may have negative effects on the mental health of young people. For example, a study of Canadian adolescents found that social media use increased after the inception of the pandemic relative to pre-pandemic usage. In addition, social media use after the pandemic began was positively correlated with depression, but social media use before the pandemic was not (Ellis et al., 2020). However, social media use was not related to self-reported loneliness.

Although the research just reviewed generally finds positive relations between social media usage and loneliness, other research has found negative relations in certain contexts. Pittman and Reich (2016) compared different kinds of social networks (image-based vs. text-based) and their impact on variables such as loneliness, happiness, and life satisfaction. They found that participants' postings on image-based platforms (e.g., Instagram, Snapchat), which they characterized as more intimate than text-based platforms (e.g., Twitter, Yik Yak), were associated with decreased feelings of loneliness and increased happiness and life satisfaction, but posting on the text-based platforms was unrelated to loneliness, happiness, and life satisfaction.

Other research has investigated how social media platforms are used, and whether type of usage affects loneliness. Perhaps unsurprisingly, positive attitudes toward social media platforms such as Instagram and Twitter are associated with lower levels of loneliness (Pittman, 2015). More interesting is the finding that how young people use the social media platforms appears to matter. Creating and consuming content, observation, and social interaction on Instagram are associated with lower levels of loneliness (Pittman, 2015; Yang, 2016). However, sharing content is associated with higher levels of loneliness. Thus, it appears that more passive activities such as interacting and observing may decrease feelings of loneliness, whereas more active–but *noninteractive*–activities such as sharing content may increase feelings of loneliness.

Summarizing, although research on the relation between social media usage and loneliness has not always been consistent, the predominant view seems to be that, overall, social media usage for adolescents and young adults is positively correlated with loneliness because of its noninteractive nature.

#### Messaging and Voice Over IP (VoIP) Apps Usage and Loneliness

One potential explanation for the inconsistencies found between social network usage and loneliness might be that interactive messaging and Voice over IP (VoIP) apps, such as WhatsApp or iMessage, are commonly examined together with less interactive social networking apps such as Facebook or Instagram. For example, research on problematic smartphone use typically focuses on the negative consequences of seeking social reassurance without clearly distinguishing between active bi-directional communication and passive checking on what is happening in one's social network (Elhai et al., 2017, 2020). Furthermore, when examining the items used in scales that are commonly used to measure smartphone problematic use, such as the smartphone addiction scale (Kwon et al., 2013; Harris et al., 2020) or the smartphone addiction inventory (Pavia et al., 2016), it is clear that most of the negative consequences found in studies that use them are driven by social networking app usage rather than communication app usage (e.g., “Constantly checking my smartphone so as not to miss conversations between other people on Twitter or Facebook.”). Additionally, these inconsistent effects are found in pre-pandemic contexts when smartphone usage, in terms of both social networking and messaging apps, interferes with face-to-face interactions (e.g., “I find myself indulged on the smartphone at the cost of hanging out with friends.”). Our research addresses these issues by examining smartphone app usage in a specific setting when there is virtually no face-to-face interaction to be disrupted (the COVID-19 pandemic setting) and by distinguishing between social networking apps and communication apps.

We posit that because of their interactive nature, messaging and VoIP app usage will be associated with reduced feelings of loneliness, results that are opposite of those for social networking app usage. Although there is scarce pre-existing literature that distinguishes between the two, we can base our prediction on research examining different typologies of social network usage that has focused on differentiating between the effects of interactive and noninteractive social network use. For example, Burke et al. (2010) distinguished between two types of activities: consumption and direct communication. Consumption refers to observing friends' conversations with others, their status updates, and their “likes.” Direct communication refers to direct interactions, such as photo tagging and messaging, between focal users and their friends. Direct communication is positively associated with relationship quality, and specific direct communications such as one-on-one chat sessions are associated with lower levels of loneliness and depression. In contrast, consumption is negatively associated with social capital and increases in feelings of loneliness.

Yang (2016) also argues that the likely cause of inconclusive findings on the link between social network usage and loneliness depends on the type of social networks usage, which can be classified into three categories: passive, active, and interactive. Passive activities are ones in which users consume or browse the content of the social network (e.g., scrolling), and these activities typically increase loneliness and decrease well-being. Active usage refers to the production of content that is not targeted to anyone in particular (e.g., updating the status of one's social network without tagging a friend), and preliminary findings relate it to higher levels of loneliness. Finally, social network activities can be interactive, meaning that users can interact and socialize directly with other users (e.g., sending direct messages), and these activities have been shown to be the only ones that are negatively correlated with loneliness. Yang (2016) further argues that even though social networks could be a channel to find support from other people, many times indirect communication hinders the ability of users to respond to content that has not been addressed specifically to them, which would not be the case if users would engage in interactive rather than merely active activities.

In sum, when there is direct communication between focal users and their friends, and when digitally mediated communication is interactive, loneliness and its associated negative consequences are attenuated, whereas when the communication is indirect, such as when focal users only observe the interaction that is taking place in their network, feelings of loneliness increase. In the present research, we focus on smartphone apps usage, and given that we are interested in determining its effect on users' loneliness, we distinguish between applications that are interactive by definition, such as WhatsApp or iMessage (i.e., messaging/VoIP communication apps), and applications that are designed to foster active and passive–but not necessarily interactive–usage, such as Facebook and Instagram (i.e., social networking apps). Furthermore, we posit that the use of messaging and VoIP apps will be associated with reduced feelings of loneliness, whereas the use of social networking apps will be associated with greater loneliness.

#### Social Network Usage, Fear of Missing Out (FOMO), and Loneliness

The research we have reviewed thus far suggests a robust positive relation between social network usage and loneliness, which raises the critical question of what drives this presumed effect? Given that the difference between the consequences of messaging apps and social networking apps is likely to be determined by their interactive and noninteractive nature, it is plausible that the negative consequences of noninteraction are driven by a perceived lack of inclusion. Specifically, when focal users observe other members of their network interacting with each other, they may feel left out of the social interaction they are passively observing. In turn, this distress might prompt them to actively share content with their network, but, if they do not get a response, they may feel even worse.

One factor that relates to feelings of being left out is *fear of missing out*, commonly referred to as FOMO. Przybylski et al. (2013, p. 1841) describe FOMO as “a pervasive apprehension that others might be having rewarding experiences from which one is absent,” and manifests as a “desire to stay continually connected with what others are doing.” Given that social networks specifically enable users to stay connected with what others are doing, then it is likely that social network usage and FOMO would be positively correlated, and several studies support this reasoning. For example, social network addiction is positively related to FOMO (Franchina et al., 2018; Gezgin, 2018), and this relation is mediated by feelings of envy (Yin et al., 2019). Similarly, Dempsey et al. (2019) found that scores on a Facebook addiction scale were positively correlated with social anxiety and negatively correlated with life satisfaction in a sample of college undergraduates, and this relation was mediated by FOMO. Specifically, Facebook addiction positively predicted FOMO, which in turn was associated with higher levels of social anxiety and decreased life satisfaction.

The positive relation between social network usage and FOMO is not only confined to problematic (addictive) social network usage, frequency of social network usage in general is also positively correlated with FOMO (Varga et al., 2016; Yin et al., 2019; Serrano, 2020). For example, Buglass et al. (2017) found that social network usage was positively associated with decreased self-esteem, and this relation was mediated by FOMO. However, it is important to note that these presumed effects of social network usage primarily apply to passive usage (e.g., scrolling), as opposed to active usage (e.g., uploading content). Although not all use of social networking apps is passive, researchers have argued that most of the features that make up their design, such as content personalization, notifications and alerts, as well as content that expires after a set amount of time (e.g., Instagram stories displayed only for 24 h), do encourage compulsive checking that triggers and sustains feelings of FOMO (Alutaybi et al., 2018, 2020). One explanation for these relations is that social network use exacerbates FOMO because social media users can modify the way other people see their profiles, and social media users strive to present a perfect image of who they are for self-presentation and impression management (Crabtree and Pillow, 2018). Activities such as censoring, exaggerating, or even lying about people's lives through the creation of online content could produce FOMO, which in turn would make others uncomfortable or envious (Jordan et al., 2011; Chou and Edge, 2012; Berezan et al., 2020). In fact, research suggests that heavy social media users are more likely to display high levels of FOMO because of their constant monitoring of what their friends are doing (Buglass et al., 2017).

FOMO has also been linked to higher levels of loneliness. For example, Barry and Wong (2020) found that FOMO positively predicted loneliness, and this relation held for both teenagers and adults, and also held for both FOMO with close friends and with family members. In another study, frequency of social media usage was positively associated with both FOMO and loneliness (Barry et al., 2017). Bernard (2020) found similar relations, and also found that loneliness and FOMO were positively correlated. One explanation for these interrelations among social network usage, FOMO, and loneliness is that individuals engage in social comparisons based on the information they see on social networks, which triggers the belief that their friends are getting involved in some event and are happier, which then evokes feelings of envy and loneliness. They are likely to feel some kind of envy from their peers, feel less connected, and suffer the fear of being left out (Wang et al., 2019; Yin et al., 2019).

## Overview of the Research

The current research had several objectives. Broadly, we wanted to determine the extent to which pandemic-induced forced isolation is associated with increased social network usage, and whether social network usage in turn is associated with certain aspects of mental health, with a particular focus on young people. Thus, we restricted our study to an age cohort commonly referred to as Centennials, or Generation Z, which is roughly those born after 1995. This age cohort is the first generation that has never known a world without the internet, and according to global surveys conducted before the pandemic, is the most frequent user of social network worldwide. For example, as of 2019, the average daily social network usage of internet users worldwide amounted to 136 min a day (DataReportal, 2019), with Centennial users averaging 175 min a day (GlobalWebIndex, 2019), of which 95% is spent on mobile devices rather than on personal computers or laptops (Statista, 2019).

First, we wanted to determine whether pandemic-related forced isolation (lockdowns) relates to social network usage. We expect that social networking app usage and communication app usage will increase during lockdowns relative to pre-lockdown usage levels, then will stabilize over time. Second, in terms of social network usage effects on aspects of mental health, we focused specifically on FOMO and loneliness. Based on theory and previous research, we test a conceptual model in which social network usage positively predicts FOMO, which in turn positively predicts loneliness. Modeling FOMO as the mediator is consistent with previous research (Buglass et al., 2017; Dempsey et al., 2019). Although our model implies theoretical causal relations, we acknowledge that not only can our cross-sectional data not determine causality, but also that the relations between all three variables are likely bi-directional. However, modeling social network usage as the independent variable is consistent with experimental findings that limiting social network usage reduces loneliness (Hunt et al., 2018). Finally, we looked at the relationship between messaging and VoIP apps with FOMO and loneliness to test whether they do indeed have opposite influences compared to social networking apps. To do so, we test a model in which communication apps have a direct negative relationship with loneliness that is not mediated by FOMO, as it is in the case of social network apps.

## Materials and Methods

### Sampling Plan, Participants, and Procedure

**Figure 1:**
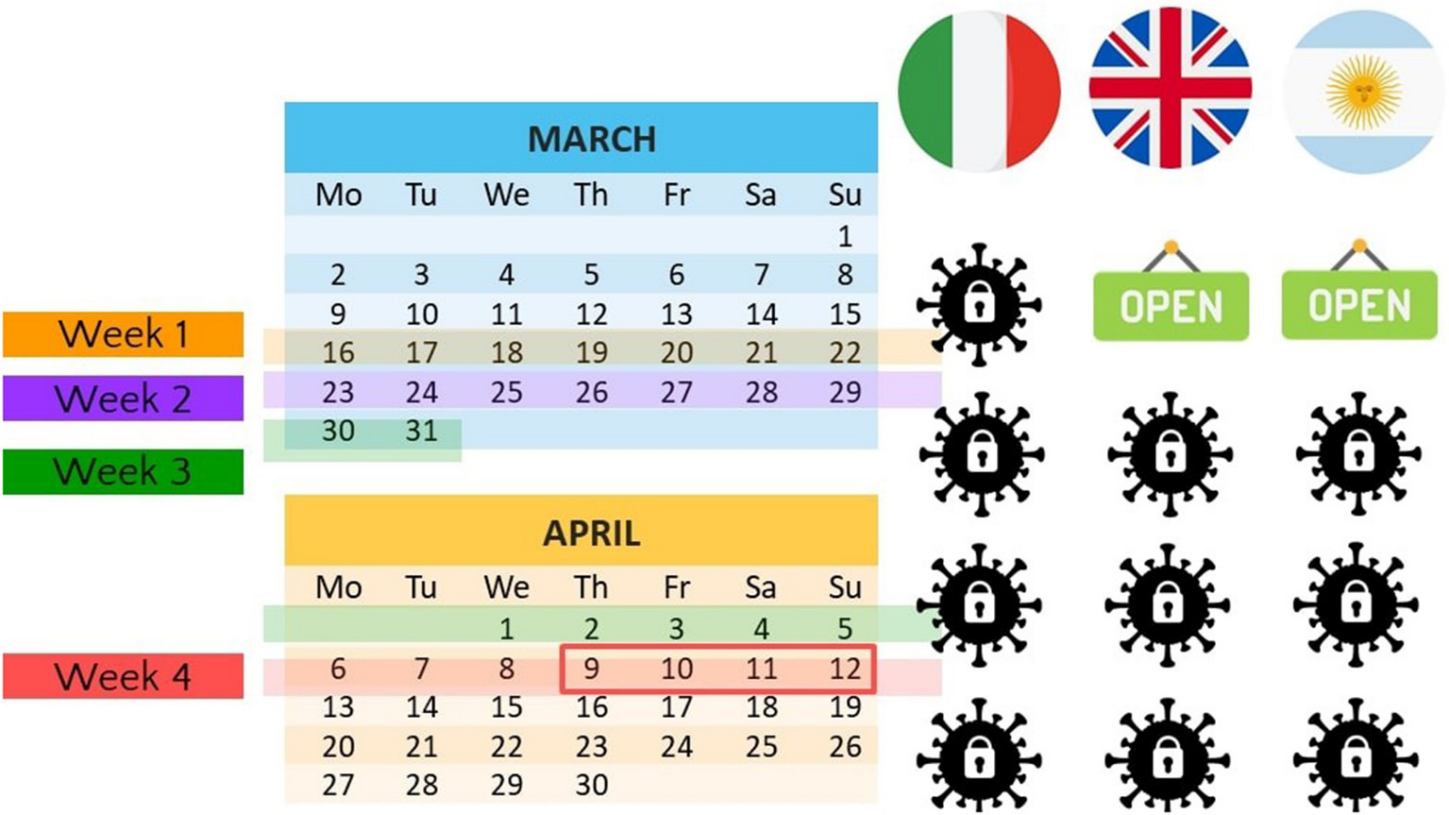
Data collection timeframe and lockdown enforcement by country.

On April 9th, 2020, we launched an online survey across three countries in which shelter-in-place orders where enforced at different times (see Figure 1): Italy (initiation of shelter-in-place March 11th), Argentina (March 20th), and the United Kingdom (March 23rd). Data for Italy, and the UK were collected on Prolific Academic; data for Argentina were collected with a sample of students. We stopped data collection on April 12th because, starting from the following day, participants could no longer provide information on Week 1 because iOS devices only display 4 weeks of data at any given time. As Figure 1 shows, screen time data collected corresponds to periods of enforced nationwide lockdowns (or not), depending on the country of focus. In particular, data from Week 1 were fundamental because it enabled us to compare before and after lockdown usage for two countries (UK and Argentina).

Using different countries with different shelter-in-place starting points enabled us to test more accurately whether the imposed isolation is associated with increased social networking and messaging/VoIP apps usage. Initially, we also collected data from a fourth country, the United States. However, a lack of a coordinated national response made it difficult to test our hypotheses given the variability of lockdown measures enforcement at both the state level and county level (please refer to Supplementary Material for more information on US lockdown enforcement and related secondary data). Thus, we decided to focus on the three countries that enabled us to test our hypotheses with a clear-cut time frame of lockdown enforcement. All data we collected, including the US sample, are available at https://osf.io/29ks6/?view_only=992f678baee14621b7dcac44c2b1f457.

**Figure 2:**
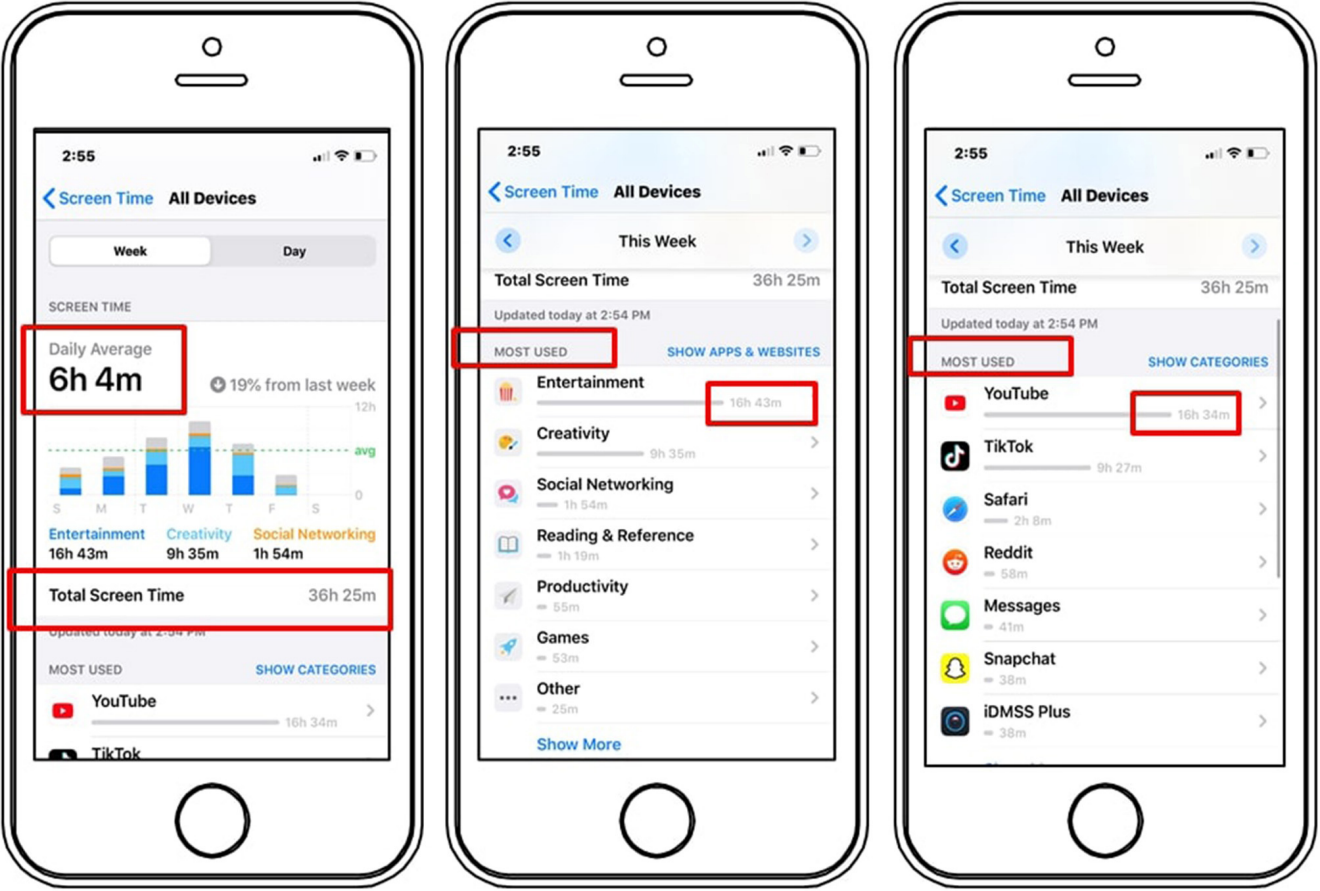
Example of weekly smartphone data coding.

Because our research was focused on younger adults, and one of our objectives was to obtain more objective measures of time spent on social media usage, we restricted participation to participants who were born after 1995 and had an iOS smartphone, which allowed us to measure screen time usage from screenshots (see Figure 2). Our final sample comprised 334 respondents (*M*_age_ = 21.50 years, *SD* = 2.03, 30.20% men), with the breakdown by country as follows: Italy (*n* = 89, *M*_age_ = 22.17, *SD* = 1.89, 39.33% men), UK (*n* = 149, *M*_age_ = 21.04, *SD* = 2.16, 26.17% men), and Argentina (*n* = 96, *M*_age_ = 21.52, *SD* = 1.77, 28.12% men). All participants provided informed consent.

The study consisted of two parts. First, participants uploaded screenshots of their phone's screen time weekly report for the 4 weeks they had available on their devices (Week 1: March 16th to 22nd; Week 2: March 23rd to 29th; Week 3: March 30 to April 5th; Week 4: April 6th to April 12th). Participants then completed a survey that measured the focal constructs. The survey was completed between April 9th and 12th, which corresponded to the middle and final part of Week 4. Thus, it is important to note that smartphone data usage collected in Week 4 varies in completeness depending on which day the participant took the survey. For example, if a participant took the survey on April 9th (Thursday of Week 4), her data for Week 4 will be incomplete, compared to another participant who took the survey on April 12th (Sunday of Week 4). Therefore, as we will explain in the Results section, when analyzing the relationship between our survey data (collected on Week 4) and social network usage, we will use smartphone data from the first 3 weeks because they provide comparable data for all participants regardless of the day on which they took the survey.

### Measures

The focal constructs we measured in Week 4's survey were FOMO, loneliness, personality traits, and demographics, in that order. All constructs were measured along 5-point scales, except for the personality variables, which were measured along 7-point scales, and composite variables for each construct were computed by averaging across item scores for each scale. All items for each scale are provided in the Supplementary Material. We measured loneliness with the 8-item short version of the revised UCLA Loneliness Scale (Hays and DiMatteo, 1987; α = 0.82). We measured personality traits with the 10-item TIPI scale, which is comprised of five factors (extroversion, emotional stability, openness to experience, agreeableness, and conscientiousness; Gosling et al., 2003). The personality traits were included to serve as covariates, because research has shown that they are related to social media use (Montag et al., 2015; Nowland et al., 2018; Kircaburun et al., 2020). We measured FOMO with a 3-item measure adapted from the original scale 10-item scale (Przybylski et al., 2013) to reflect COVID-specific circumstances. Specifically, we asked participants to rate their agreement with three statements (“I'm worried my friends will have video chats without me,” “I wonder if I spend too much time on my phone trying to keep up with what is going on,” “When I have a good time it is important for me to share the details online (e.g., updating status),” along a 5-point scale (1 = strongly disagree, 5 = strongly agree). The three items were selected to reflect the different components of FOMO (worry that others are having experiences without them and the desire to stay continually connected to what others are doing). The Cronbach's alpha was low (α = 0.39), which may partly reflect the small number of items. The inter-correlations were relatively uniform and all corrected item-total correlations exceeded the accepted cut-off of 0.30 (Nunnally, 1994), so we retained all three items for analyses.

#### Screen Time Usage Measures

After the data were collected, we coded smartphone screenshots so that we could pair survey responses with smartphone usage data. From the screenshots, we extracted and coded: (1) total time spent using the phone (e.g., total weekly screen time), (2) usage time of different app categories (e.g., Social Network, Productivity), and (3) usage time of single apps installed (e.g., Instagram, WhatsApp). We also coded whether participants had set usage limits for some of their apps (0 = no limits set, 1 = limits set). In addition, given that in our hypothesized relationships we distinguish social networks from communication apps, we computed two additional variables by summing all single social network apps (e.g., Facebook, Instagram, Snapchat, TikTok) and all messaging and VoIP apps (e.g., WhatsApp, iMessage, Zoom, Facebook Messenger), rather than relying on what iOS reports as “social network category,” because the latter includes both types of apps. (For more information on how Apple developers are prompted to classify apps by category, please see https://developer.apple.com/app-store/categories/.).

## Results

Demographic information by country and descriptive statistics for the main variables are provided in Tables 1, 2. As Table 2 shows, there were country differences regarding scores on several of the variables. However, these differences did not fundamentally affect the relations between the variables. Thus, we collapsed the data across country for the critical hypothesis-testing.

**Table 1:** Sample demographic information and descriptive statistics by Country.

	**Argentina (*n* = 96)**	**Italy (*n* = 89)**	**UK (*n* = 149)**	**Overall (*n* = 334)**
**Age**				
Mean (SD)	21.5 (1.77)	22.2 (1.89)	21.0 (2.16)	21.5 (2.03)
Median (Min, Max)	22.0 (18.0, 25.0)	22.0 (18.0, 26.0)	21.0 (18.0, 26.0)	22.0 (18.0, 26.0)
**Gender**				
Female	69 (71.9%)	53 (59.6%)	109 (73.2%)	231 (69.2%)
Male	27 (28.1%)	35 (39.3%)	39 (26.2%)	101 (30.2%)
Other	0 (0%)	1 (1.1%)	1 (0.7%)	2 (0.6%)
**FOMO**				
Mean (SD)	2.20 (0.812)	2.21 (0.809)	2.47 (0.740)	2.32 (0.789)
Median (Min, Max)	2.17 (1.00, 4.33)	2.00 (1.00, 4.33)	2.67 (1.00, 5.00)	2.33 (1.00, 5.00)
**Loneliness**				
Mean (SD)	2.05 (0.490)	2.49 (0.620)	2.52 (0.610)	2.38 (0.615)
Median (Min, Max)	2.00 (1.00, 3.25)	2.38 (1.38, 4.63)	2.50 (1.25, 5.00)	2.25 (1.00, 5.00)
**Personality Trait - Extroversion**				
Mean (SD)	4.95 (1.29)	3.44 (1.63)	4.32 (1.49)	4.27 (1.58)
Median (Min, Max)	5.00 (1.00, 7.00)	3.00 (1.00, 7.00)	4.50 (1.00, 7.00)	4.50 (1.00, 7.00)
**Personality Trait - Emotional Stability**				
Mean (SD)	3.86 (1.35)	4.21 (1.46)	4.12 (1.42)	4.07 (1.41)
Median (Min, Max)	3.50 (1.00, 7.00)	4.50 (1.50, 7.00)	4.00 (1.50, 7.00)	4.50 (1.00, 7.00)
**Personality Trait - Openness to Experience**				
Mean (SD)	5.11 (1.07)	4.84 (1.17)	4.82 (1.09)	4.91 (1.11)
Median (Min, Max)	5.50 (2.50, 7.00)	5.00 (2.00, 7.00)	5.00 (1.50, 7.00)	5.00 (1.50, 7.00)
**Personality Trait - Agreeableness**				
Mean (SD)	4.80 (0.975)	4.84 (1.05)	4.83 (1.17)	4.82 (1.08)
Median (Min, Max)	4.50 (2.50, 7.00)	5.00 (2.50, 7.00)	4.50 (1.50, 7.00)	4.50 (1.50, 7.00)
**Personality Trait - Conscientiousness**				
Mean (SD)	5.19 (1.22)	5.15 (1.26)	5.02 (1.35)	5.10 (1.29)
Median (Min, Max)	5.25 (2.00, 7.00)	5.50 (2.00, 7.00)	5.50 (1.50, 7.00)	5.50 (1.50, 7.00)
**Average Weekly Social Networking Apps Usage (3 weeks, hours)**				
Mean (SD)	23.5 (10.0)	18.0 (10.9)	24.9 (13.8)	22.7 (12.3)
Median (Min, Max)	23.6 (0.0583, 48.4)	17.8 (0, 50.4)	23.0 (0, 64.1)	21.3 (0, 64.1)
Missing	2 (2.1%)	9 (10.1%)	15 (10.1%)	26 (7.8%)
**Average Weekly Messaging and VoIP Apps Usage (3 weeks, hours)**				
Mean (SD)	14.0 (6.57)	11.6 (7.81)	5.42 (4.72)	9.65 (7.29)
Median (Min, Max)	12.6 (0.778, 29.3)	9.97 (0.983, 47.7)	4.15 (0, 28.0)	8.46 (0, 47.7)
Missing	2 (2.1%)	9 (10.1%)	15 (10.1%)	26 (7.8%)

**Table 2:** Main variables, descriptive statistics and correlations.

**Variable**	***M***	***SD***	**1**	**2**	**3**	**4**	**5**	**6**	**7**	**8**	**9**	**10**
1. Loneliness	2.38	0.62										
2. Social networking apps usage	22.67	12.32	0.10									
3. Messaging and VoIP apps usage	9.65	7.29	−0.30^***^	−0.08								
4. FOMO	2.32	0.79	0.26^***^	0.19^***^	−0.03							
5. Extroversion	4.27	1.58	−0.55^***^	0.04	0.10^**^	0.04						
6. Emotional stability	4.07	1.41	−0.35^***^	−0.08	0.04	−0.22^***^	0.04					
7. Agreeableness	4.82	1.08	−0.15^**^	−0.03	−0.02	−0.11^*^	−0.04	0.20^***^				
8. Openness to experience	4.91	1.11	−0.33^***^	−0.13^*^	0.16^**^	−0.03	0.36^***^	0.14^**^	0.05			
9. Conscientiousness	5.10	1.29	−0.14^**^	−0.14^*^	0.09	−0.12^**^	0.02	0.18^***^	0.10^**^	0.06		
10. Age	21.48	2.03	−0.03	−0.25^***^	0.20^***^	−0.08	−0.09	0.12^*^	0.03	0.02	0.07	
11. Gender	0.30	0.46	−0.02	−0.00	−0.02	−0.15^**^	−0.15^**^	0.21^***^	−0.15^**^	−0.07	−0.00	0.05

*M and SD are used to represent mean and standard deviation, respectively. Gender is coded as 1 = Male; 0 = Female/Other*.

* *indicates p < 0.05*.

** indicates p < 0.01

*** *indicates p < 0.001*.

### Social Network and Messaging Apps Usage as a Function of Lockdown Initiation

We first tested whether social network usage increased based on whether a nationwide lockdown was initiated during the weeks we collected data for the different countries we sampled. Note that, as mentioned in the data collection procedure description, because participants took the survey between April 9th and 12th, we did not use data for Week 4 because not all participants had the same volume of data (i.e., some had 3 days, some had four). Thus, we compared social network usage between the first 3 weeks of complete smartphone data. To do so, we conducted a two-way mixed-model ANOVA using the lme4 R package (Bates et al., 2014), with weekly average usage as a within-subjects factor and country of residence as a between-subjects factor. As expected, the main effect of week of usage on social network usage was significant [*F*_(2,527)_ = 24.91, *p* < 0.001, η2 = 0.08)]. Participants' spent less time on social network apps in the earliest week recorded compared to the subsequent 2 weeks when the pandemic evolved and lockdowns began to be enforced in more countries (see Table 3, panel A).

**Table 3:** Mixed-model ANOVA results for social networking app usage.

**Estimated Marginal Means (A)**		**Estimated Marginal Means (B)**		**Estimated Marginal Means (C)**			
**Mean (SE)**		**Mean (SE)**		**Mean (SE)**			
Week 1	19.9 (0.77)^a^	Argentina	23.3 (1.23)^a^		Argentina	Italy	UK
Week 2	22.8 (0.76)^b^	Italy	18.0 (1.33)^b^	Week 1	21.0 (1.37)^a^	16.7 (1.48)^a^	22.0 (1.15)^a^
Week 3	23.3 (0.76)^b^	UK	24.7 (1.03)^a^	Week 2	25.0 (1.34)^b^	18.3 (1.45)^a^	25.2 (1.13)^b^
				Week 3	24.0 (1.33)^b^	18.9 (1.45)^a^	27.0 (1.12)^b^

*Means sharing the same superscript are not significantly different from each other*.

The main effect of country was also significant [*F*_(2,300)_ = 8.59, *p* < 0.001, η2 = 0.05)]. Participants from Italy spent significantly less time on social networks (~4–6 h less per week) compared to those from the other two countries (see Table 3). More direct to our hypothesis, the interaction between week and country was marginally significant [*F*_(4,526)_ = 2.22, *p* = 0.066, η2 = 0.02)]. To decompose the interaction, we conducted planned comparisons of social network usage within country between the different weeks (see Table 3, panel C). For Italy, as expected, no differences between weekly social network usage were found because all 4 weeks of data collected corresponded to a nationwide enforced lockdown that was already in place since March 11th. However, for Argentina and UK participants, social network usage was significantly lower in the pre-lockdown week (Week 1) compared to the following 2 weeks when a nation-wide lockdown was in place.

Next, we tested whether messaging increased based on lockdown initiation. We again conducted a two-way mixed-model ANOVA, with weekly average usage as a within-subjects factor and country of residence as a between-subjects factor. In particular, we found a main effect of week on usage of messaging and VoIP apps [*F*_(2,532)_ = 9.67, *p* < 0.001, η2 = 0.04)]. Participants' spent increasingly more time using those apps as weeks went by (see Table 4, panel A), even if substantially less (roughly half the number of hours) compared to social networking apps.

**Table 4:** Mixed-model ANOVA results for messaging and VoIP app usage.

**Estimated Marginal Means (A)**		**Estimated Marginal Means (B)**		**Estimated Marginal Means (C)**			
	**Mean (SE)**		**Mean (SE)**	**Mean (SE)**			
Week 1	8.24 (0.34)^a^	Argentina^a^	13.9 (0.59)		Argentina	Italy	UK
Week 2	8.88 (0.33)^a^	Italy^b^	11.55 (0.64)	Week 1	12.70 (0.73)^a^	11.25 (0.79)^a^	4.77 (0.62)^a^*^^
Week 3	9.58 (0.33)^b^	USA^c^	4.73 (0.57)	Week 2	13.67 (0.71)^b^	11.51 (0.77)^a^	5.80 (0.60)^a^*^^
		UK^c^	5.42 (0.50)	Week3	15.34 (0.71)^c^	11.89 (0.77)^a^	5.66 (0.60)^a^

*Means sharing the same superscript are not significantly different from each other*;

* *p = 0.084*.

The main effect of country was also significant [*F*_(2,304)_ = 57.17, *p* < 0.001, η2 = 0.28)], with large differences in messaging and VoIP apps usage. Participants from the UK spent significantly less time using these apps compared to participants from Argentina and Italy (see Table 4, panel B). In addition, similar to what we found for social networking apps, the interaction between country and week was also significant [*F*_(4,531)_ = 2.59, *p* = 0.036, η2 = 0.02)]. We expected participants' usage of messaging and VoIP apps to increase significantly between Week 1 and Week 2 in Argentina and in the UK, given that a nationwide lockdown was announced at the end of Week 1 in both countries. As predicted, usage of messaging and VoIP apps increased marginally significantly following lockdown enforcement (from Week 1 to Week 2) in the UK (*p* = 0.084), whereas it remained constant in Italy, where the nationwide lockdown was already in place before data collection began (see Table 4, panel C). In Argentina, we also found that usage increased between Week 1 and Week 2 as expected, but usage kept increasing in Week 3 as well, and thus cannot be solely explained by lockdown enforcement.

### Social Network Usage, Loneliness, and FOMO

**Figure 3:**
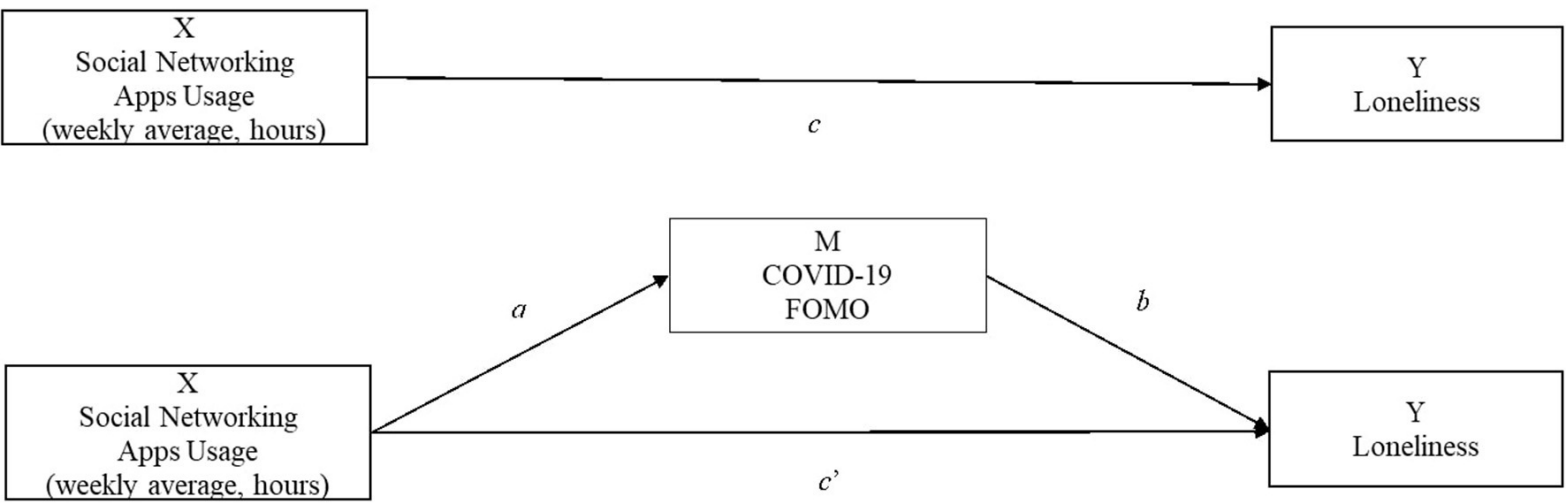
Mediation model with social network app usage as predictor.

Next, we tested our mediation model in which social network usage is positively associated with loneliness, and this relation is mediated by FOMO: social network usage is positively related to FOMO, which in turn is positively related to loneliness. We tested this mediation using the Lavaan R package (Rosseel, 2012) with 5,000 bootstrapping samples. As depicted in Figure 3, the dependent variable (Y) is participants' levels of loneliness, the independent variable (X) is social network usage, and the mediating variable (M) is participants' levels of FOMO.

The path estimates support the hypothesized model (see Table 5). Participants' average social network usage of the previous 3 weeks is positively correlated with their FOMO measured during the fourth week (path a in Figure 3: *b* = 0.012, SE = 0.004, 95% CI [0.004, 0.020]), and participants' level of FOMO is positively correlated with their level of loneliness (path b in Figure 3: *b* = 0.20, SE = 0.05, 95% CI [0.113, 0.280]). The direct relation between social network usage and loneliness is not significant (path c': *b* = 0.003, SE = 0.001, 95% CI [−0.004, 0.009]), whereas the indirect effect is (*b* = 0.002, SE = 0.001, 95% CI [0.001, 0.005]). These result support the mediation hypothesis. Finally, adding country fixed effects, personality scores and gender as covariates in the model did not change any fundamental relations (all tested models can be found in Supplementary Material).

**Table 5:** Mediation results for social network usage as predictor.

**Model-Path Estimates**				
	**Coefficient**	**SE**	**z**	***p***
**a**	**0.012**	**0.004**	**3.150**	**0.002**
**b**	**0.197**	**0.042**	**4.639**	**0.000**
c	0.005	0.003	1.532	0.125
c′	0.003	0.003	0.783	0.434
**Indirect Effect (with Bootstrap 95% Confidence Interval and Standard Errors)**				
	**Effect**	**LL 95%CI**	**UL 95% CI**	**SE**
**X → M → Y**	**0.002**	**0.001**	**0.005**	**0.001**

*5,000 bootstraps. Bolded paths are significant*.

### Messaging and Voice Over IP (VoIP) Apps Usage, Loneliness, and FOMO

**Figure 4:**
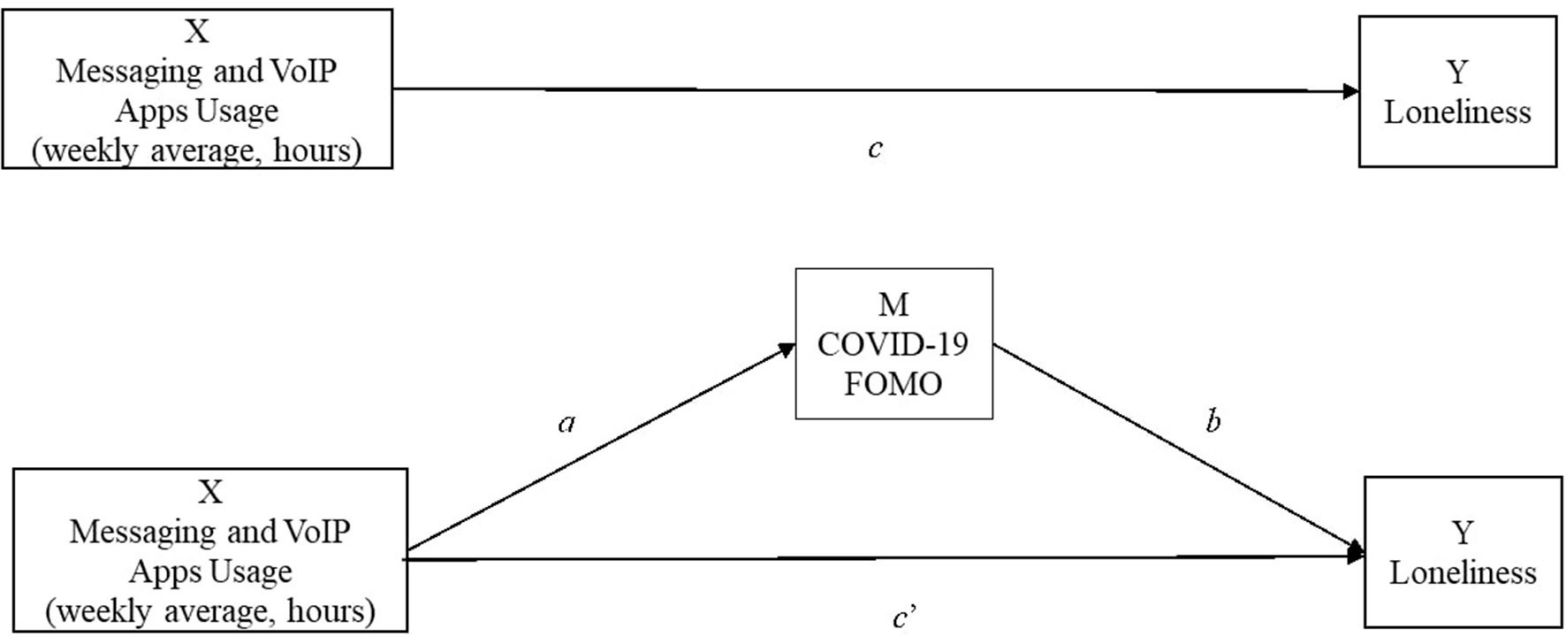
Mediation model with messaging and VoIP app usage as predictor.

To test our hypothesis that communication apps fostering a more direct form of peer-to-peer communication (e.g., WhatsApp, iMessage) will reduce loneliness while not increasing FOMO, we ran the same mediation model using the weekly average of the sum of all messaging apps as the independent variable (see Figure 4). The path estimates confirmed the hypothesized effects (see Table 6). Contrary to what we found using social networking usage as the independent variable, with communication apps, we found no indirect effect through FOMO (*b* = −0.001, SE = 0.001, 95% CI [−0.003, 0.002]), and we found a negative direct effect of communication apps usage on participants' feelings of loneliness (path c′ in Figure 4: *b* = −0.02, SE = 0.006, 95% CI [−0.035, −0.012]).

**Table 6:** Mediation results for messaging and VoIP apps as predictor.

**Model-Path Estimates**				
	**Coefficient**	**SE**	**z**	***p***
a	−0.003	0.006	−0.526	0.599
**b**	**0.199**	**0.039**	**5.050**	**0.000**
**c**	**−0.024**	**0.006**	**−4.114**	**0.000**
**c′**	**−0.024**	**0.006**	**−4.052**	**0.000**
**Indirect Effect (with Bootstrap 95% Confidence Interval and Standard Errors)**				
	**Effect**	**LL 95%CI**	**UL 95% CI**	**SE**
X → M → Y	−0.001	−0.00	0.002	0.001

*5,000 bootstraps. Bolded paths are significant*.

## Discussion

To our knowledge, this is the first empirical study to investigate the potential effects of physical isolation following COVID-19 lockdowns on Centennials' social network usage and feelings of loneliness. In particular, we collected smartphone screen time data to test whether social network usage increased during period of enforced physical distancing (lockdowns) compared to regular usage when restrictions were not in place. By comparing pre-lockdown and post-lockdown usage between countries who enforced it within our data collection period (i.e., Argentina, UK), and countries that already had it in place (i.e., Italy), we found that social network usage did increase once a nation-wide lockdown was enforced. Messaging and VoIP apps usage also increased, but seemed not to be determined solely by lockdown enforcement.

We also investigated whether increased social network usage was associated with increased of loneliness. Previous research conducted with a Canadian sample provided initial evidence that (self-reported) usage of social media increased during the pandemic, and that it correlated positively with depression but not with loneliness (Ellis et al., 2020). We add to this research by showing that social networking usage is indeed associated with higher levels of loneliness, but that the relationship is fully mediated by increased FOMO. We also offer some evidence that not all screen time has negative consequences. In fact, we show that usage of messaging and VoIP apps (e.g., WhatsApp) may help reduce feelings of loneliness because it does not influence individuals' FOMO.

Summarizing, we found that lockdown initiation affected social network app usage but not messaging and VoIP apps usage. Messaging apps usage differed markedly between countries, but even in countries that seemed to be heavy users, the number of weekly hours spent using these apps were substantially lower compared to hours spent on social network apps. Given that we showed that the latter may have a detrimental effect on young adults' mental health because of its positive correlation with both FOMO and loneliness, a possible intervention is to encourage the use of messaging and VoIP apps, while discouraging passive social network usage, during periods of physical isolation. Previous research has shown that mental well-being can be enhanced with socio-technical approaches aimed at reappraisal of FOMO (e.g., self-talk, checklists; Alutaybi et al., 2020), as well as cognitive reappraisal of time spent alone (e.g., reappraise their time alone as solitude rather than loneliness; Rodriguez et al., 2020). Thus, public policy interventions encouraging young adults to adopt approaches that help them manage negative experiences such as FOMO or perceived isolation could greatly help reduce their negative consequences on mental health, especially in highly stressful situations that trigger a compulsive use of technology.

Our research has important limitations that should be noted. First, even though the Gen Z demographic has been shown to mainly use social networking sites via their mobile devices, screen time data was collected only on smartphones, and participants could have accessed social networks from desktop or laptop computers as well. Second, based on iOS screen time data, we were unable to distinguish between social network posting/commenting and social network browsing. The two have been shown to have opposite effects on well-being and coping (Yang et al., 2020), and future research would profit from untangling these behaviors, and with data other than self-report. Third, this survey was completed at the beginning of April, and there is no baseline for comparison of pre-pandemic loneliness levels. However, we examined the impact of smartphone usage that was recorded by the device 3 weeks before participants reported their levels of loneliness.

Another limitation is that our sample is predominantly female. Recent research suggests that women may be more lonely than men (Rönkä, 1986; Beutel et al., 2017). Moreover, loneliness is often associated with factors that affect women more negatively than men, such as infertility (Jirka et al., 1996; Repokari et al., 2007) or living without a partner (Beutel et al., 2017). Thus, even though our results remain unchanged when adding gender as a covariate, the magnitude of some findings may be greater than would be observed in populations in which gender is more balanced.

An additional issue that limits the interpretation of our findings is that we did not collect information about the pre-pandemic in-person social networks of the participants, which could have been useful to see how the lockdown affected in-person compared to digital social networks. That said, research suggests that digital social networks normally mirror in-person social networks, because online tools are usually used to strengthen different aspects of people's offline connections (Subrahmanyam et al., 2008) and offline identities (McMillan and Morrison, 2006). Additionally, even though we do not have the specifics of their offline social relationships before or during the pandemic, we obtained their screen time usage data over a 4-week period that provided information about how they used digital social networks before lockdowns. Nevertheless, when interpreting the results of this study, it is important to consider that both online and offline dimensions of social networks are fundamental (Kwak and Kim, 2017), and we lack information regarding one of those.

Finally, this research only proposed a possible intervention that might need to be further explored in a post-pandemic context. It is be plausible that usage of messaging and VoIP apps increase FOMO when messaging competes with real-life events.

## Data Availability Statement

The datasets presented in this study can be found in online repositories. The names of the repository/repositories and accession number(s) can be found below: https://osf.io/29ks6/?view_only=992f678baee14621b7dcac44c2b1f457.

## Ethics Statement

Ethical review and approval was not required for the study on human participants in accordance with the local legislation and institutional requirements. The patients/participants provided their written informed consent to participate in this study.

## Author Contributions

EF: research design, data processing, and manuscript writing. LS: manuscript writing and revision. MD: data collection and processing. All authors contributed to the article and approved the submitted version.

## Conflict of Interest

The authors declare that the research was conducted in the absence of any commercial or financial relationships that could be construed as a potential conflict of interest.
