# Supplementary material for: Centennials, FOMO, and Loneliness: An Investigation of the Impact of Social Networking and Messaging/VoIP Apps Usage During the Initial Stage of the Coronavirus Pandemic
Source: Front Psychol. 2021 Feb 9;12:620739. doi: 10.3389/fpsyg.2021.620739 (PMC7900425; doi:10.3389/fpsyg.2021.620739)
Supplement: Supplementary file 1 [file Data_Sheet_1.PDF]

UCLA Loneliness Measure (Short 8-item version; Roberts, Lewinsohn, and Seeley 1993)

For each statement below, please indicate how often you feel the way described.

1. I feel in tune with people around me. (R)
2. I lack companionship.
3. I am an outgoing person. (R)
4. I feel left out.
5. I feel isolated from others.
6. I can find companionship when I want to. (R)
7. I am unhappy being so withdrawn.
8. People are around me but not with me.

1 = Never; 2 = Rarely; 3 = Sometimes; 4 = Most of the time; 5 = Always

COVID-19 FOMO (Adapted from Przybylski, A. K., Murayama, K., DeHaan, C. R., & Gladwell, V. 2013)

1. I'm worried my friends will have video chats without me.
2. I wonder if I spend too much time on my phone trying to keep up with what is going on.
3. When I have a good time it is important for me to share the details online (e.g. updating status).

1 = Strongly Disagree; 5 = Strongly Agree.

Ten-item personality measure (TIPI; Gosling, S. D., Rentfrow, P. J., & Swann, W. B. 2003).

Here are a number of personality traits that may or may not apply to you. Please write a number next to each statement to indicate the extent to which you agree or disagree with that statement. You should rate the extent to which the pair of traits applies to you, even if one characteristic applies more strongly than the other.

1 = Disagree strongly; 7 = Agree strongly

I see myself as:

1. _____ Extraverted, enthusiastic.
2. _____ Critical, quarrelsome.
3. _____ Dependable, self-disciplined.
4. _____ Anxious, easily upset.
5. _____ Open to new experiences, complex.
6. _____ Reserved, quiet.
7. _____ Sympathetic, warm.
8. _____ Disorganized, careless.
9. _____ Calm, emotionally stable.
10. _____ Conventional, uncreative.

TIPI scale scoring ("R" denotes reverse-scored items):
Extraversion: 1, 6R; Agreeableness: 2R, 7; Conscientiousness; 3, 8R; Emotional Stability: 4R, 9; Openness to Experiences: 5, 10R.

Sample Demographic Information

	Argentina (n=96)	Italy (n=89)	UK (n=149)	Overall (n=334)
**Age**				
Mean (SD)	21.5 (1.77)	22.2 (1.89)	21.0 (2.16)	21.5 (2.03)
Median [Min, Max]	22.0 [18.0, 25.0]	22.0 [18.0, 26.0]	21.0 [18.0, 26.0]	22.0 [18.0, 26.0]
**Gender**				
female	69 (71.9%)	53 (59.6%)	109 (73.2%)	231 (69.2%)
male	27 (28.1%)	35 (39.3.1%)	39 (26.2%)	101 (30.2%)
other	0 (0%)	1 (1.1%)	1 (0.7%)	2 (0.6%)
**Do you have a job?**				
No	47 (49.0%)	61 (68.5%)	66 (44.3%)	174 (52.1%)
Yes, part-time	38 (39.6%)	18 (20.2%)	37 (24.8%)	93 (27.8%)
Yes, full-time	11 (11.5%)	10 (11.2%)	46 (30.9%)	67 (20.1%)
**Do you have a pet?**				
No	45 (46.9%)	44 (49.4%)	62 (41.6%)	151 (45.2%)
Yes	51 (53.1%)	45 (50.6%)	87 (58.4%)	183 (54.8%)
**Living Situation**				
With someone	93 (96.9%)	89 (100%)	141 (94.6%)	323 (96.7%)
Alone	3 (3.1%)	0 (0%)	8 (5.4%)	11 (3.3%)

Mediation Model (Social Networking Apps Usage) with Country Fixed Effects, Personality Scores and Gender as Covariates

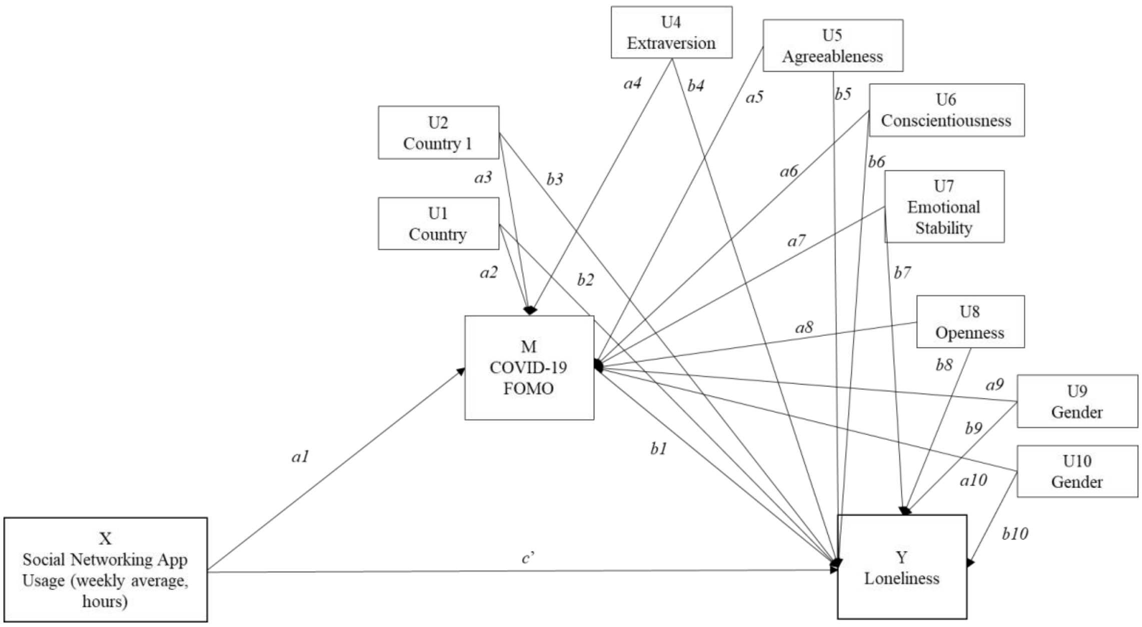

Model Results

MEDIATION RESULTS

Model-Path Estimates				
	Coefficient	SE	z	*p*
**a1**	**.0010**	**.0004**	**2.416**	**.016**
a2	.102	.123	.834	.404
**a3**	**.309**	**.123**	**2.939**	**.003**
a4	.027	.033	.824	.410
a5	-.029	.043	-.668	.504
a6	-.022	.041	-.542	.588
**a7**	**-.122**	**.031**	**-3.897**	**.000**
a8	.027	.049	.551	.582
a9	-.181	.095	-1.902	.057
**a10**	**.868**	**.343**	**2.532**	**.011**
**b1**	**.131**	**.030**	**4.289**	**.0000**
**b2**	**.147**	**.069**	**2.131**	**.033**
**b3**	**.350**	**.056**	**6.280**	**.0000**
**b4**	**-.185**	**.019**	**-9.793**	**.0000**
**b5**	**-.067**	**.021**	**-3.166**	**.002**
b6	-.013	.019	-.667	.505
**b7**	**-.113**	**.018**	**-6.295**	**.0000**
b8	-.039	.024	-1.624	.104
b9	-.053	.057	-.929	.353
b10	-.246	.524	-.470	.638
c'	.0001	.002	.580	.562
Indirect Effect (with Bootstrap 95% Confidence Interval and Standard Errors)				
	Effect	LL 95%CI	UL 95% CI	SE
**X → M → Y***	**.001**	**.000**	**.003**	**.001**

Note— 5,000 bootstraps. Bolded paths are significant; * *p* = .069

Mediation Model (Messaging and VoIP Apps Usage) with Country Fixed Effects, Personality Scores and Gender as Covariates

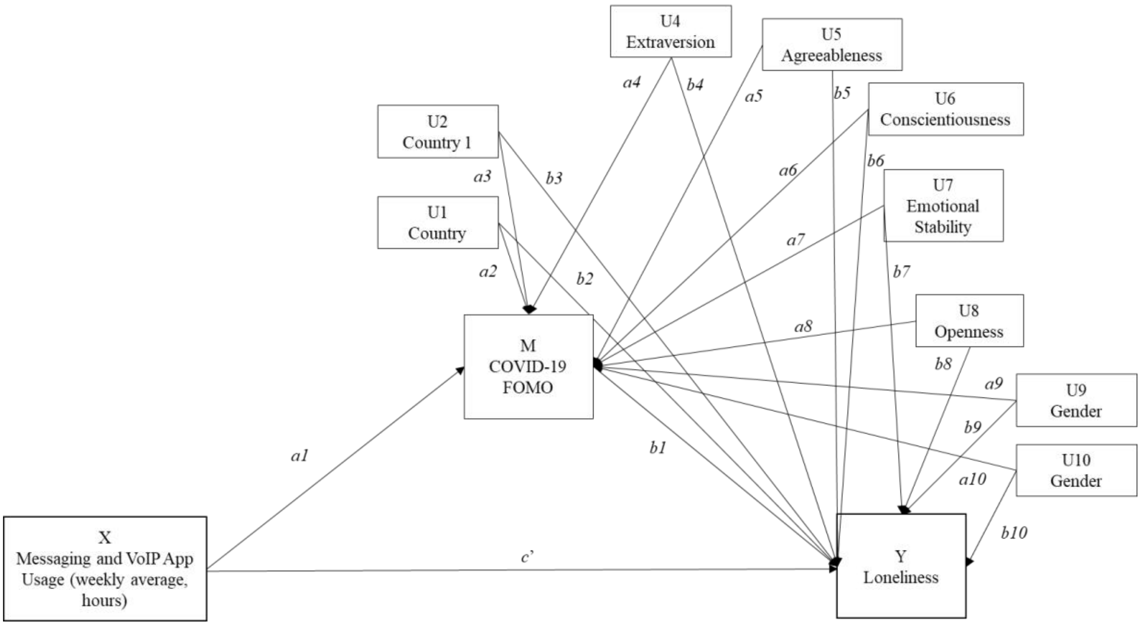

Model Results

MEDIATION RESULTS

Model-Path Estimates

	Coefficient	SE	z	*p*
a1	-.012	.007	1.800	.072
a2	.076	.121	.630	.529
**a3**	**.418**	**.1225**	**3.421**	**.001**
a4	.028	.032	.880	.379
a5	-.032	.044	-.724	.469
a6	-.036	.038	-.924	.356
**a7**	**-.127**	**.031**	**-4.061**	**.0000**
a8	.005	.049	.106	.915
a9	-.164	.096	-1.710	.087
**a10**	**.985**	**.397**	**2.481**	**.013**
**b1**	**.142**	**.031**	**4.572**	**.0000**
b2	.116	.069	1.697	.090
**b3**	**.264**	**.068**	**3.867**	**.0000**
**b4**	**-.184**	**.020**	**-9.368**	**.0000**
**b5**	**-.065**	**.021**	**-3.121**	**.002**
b6	-.012	.019	-.638	.534
**b7**	**-.109**	**.017**	**-6.352**	**.0000**
b8	-.035	.023	-1.508	.132
b9	-.057	.057	-.996	.319

b10	-.264	.494	-.534	.593
**c'**	**-.0010**	**.0004**	**-2.313**	**.021**
Indirect Effect (with Bootstrap 95% Confidence Interval and Standard Errors)				
	Effect	LL 95%CI	UL 95% CI	SE
X → M → Y*	.002	.000	.004	.001

Note— 5,000 bootstraps. Bolded paths are significant; * $p = .111$

Mediated Moderation Model - Self-Imposed Limits as Moderator (Exploratory Analyses)

Although we did not have an a priori hypothesis, we wanted to test whether having usage limits in place moderates the relationship between social media usage and FOMO. Previous research found that prompting participants to set limits for their social media usage does indeed reduce loneliness (Hunt et al., 2018). Thus, we tested whether the same effect holds for self-imposed (rather than researcher-imposed) limits, and if it applies to this specific pandemic context. Also, if limiting social networking apps reduces the negative consequences, it would suggest a possible actionable intervention to help people manage the negative effects of social network usage during this pandemic.

To test this possibility, we conducted a moderated mediation analysis (see results below). Contrary to what previous research has suggested, we found no significant interaction between social media usage and self-imposed limits on FOMO, and the overall indirect effect on loneliness was not moderated by self-imposed limits, as the index of moderated mediation was not significant. Our results could be explained by the fact that limits can still be circumvented because on the iPhone the user can continue using the app after the set time passed by ignoring the time warning, or by the fact that only a very small number of participants set self-imposed limits.

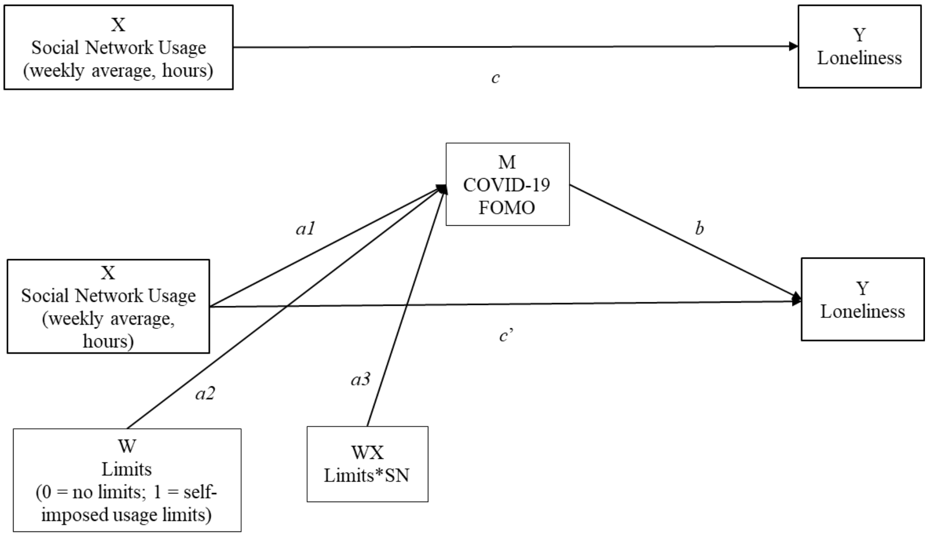

Model Results

Model-Path Estimates				
	Coefficient	SE	z	*p*
**a1**	**.012**	**.004**	**2.836**	**.005**
a2	.220	.136	1.618	.106
a3	.009	.010	0.909	.364
**b**	**.196**	**.042**	**4.653**	**.000**
c'	.003	.003	.779	.436
Indirect Effect (with Bootstrap 95% Confidence Interval and Standard Errors)				
	Effect	LL 95%CI	UL 95% CI	SE
X → M → Y				
**Without limits**	**.002**	**.001**	**.005**	**.001**
**With limits**	**.004**	**.001**	**.009**	**.002**
Index of mediated moderation				
	Index	LL 95%CI	UL 95% CI	SE
	.002	-.002	.006	.002

Note— 5,000 bootstraps. Bolded paths are significant.

Mediation Model – Single Apps as Predictors (Exploratory Analyses)

In the main manuscript, we used total social network usage as the predictor variable. However, we were also interested in examining whether the relations between smartphone usage, FOMO, and loneliness depend on the specific app used.

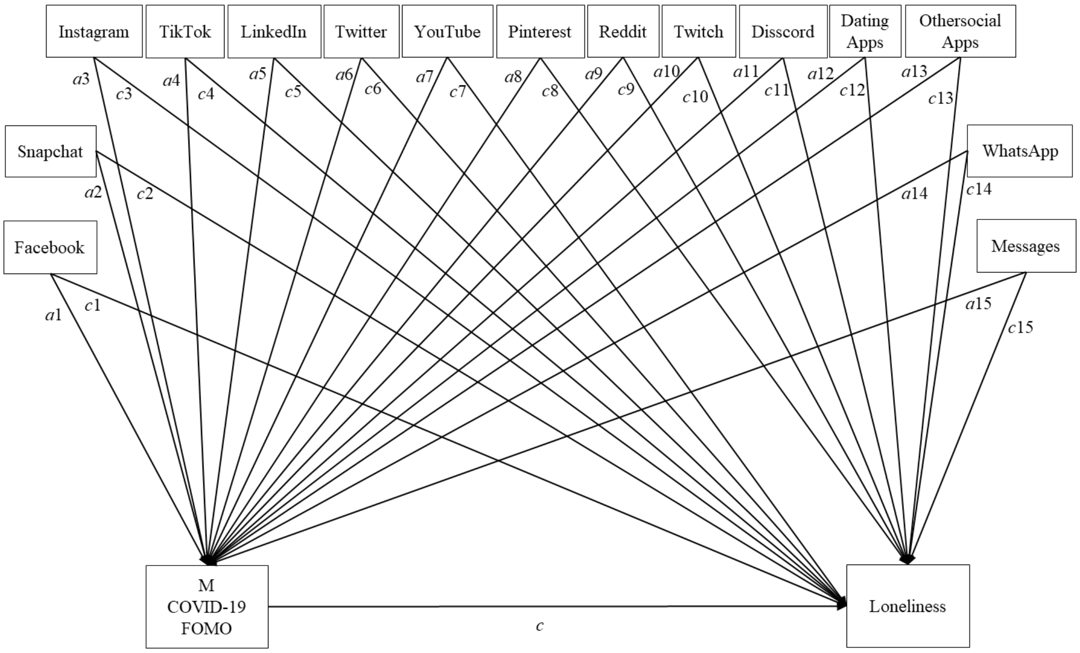

Model Results

	Model-Path Estimates			
	Coefficient	SE	z	*p*
a1	.020	.012	1.602	.109
a2	.015	.011	1.296	.195
**a3**	**.015**	**.007**	**2.245**	**.025**
a4	.013	.011	1.167	.243
a5	-.015	.271	-.054	.957
a6	.023	.015	1.463	.143
a7	-.014	.010	-1.471	.141
a8	.340	.250	1.357	.175
a9	-.026	.055	-.465	.642
a10	.031	.046	.670	.503
a11	-.011	.096	-.120	.905
a12	.094	.142	.663	.507
a13	-.017	.040	-.427	.670
a14	-.010	.008	-1.333	.182

a15	.008	.040	.201	.840
**b**	**.196**	**.041**	**4.780**	**.000**
c1	-.007	.009	.788	.431
c2	-.008	.008	-.981	.326
c3	-.006	.005	-1.171	.242
c4	-.006	.009	-.666	.506
c5	-.267	.181	-1.471	.141
c6	-.006	.008	-.767	.443
**c7**	**.013**	**.006**	**2.014**	**.044**
c8	.031	.239	.129	.897
c9	.022	.043	.518	.605
**c10**	**.089**	**.036**	**2.495**	**.013**
c11	.014	.040	0.349	.727
c12	.004	.085	.045	.964
c13	.018	.025	.739	.460
**c14**	**-.025**	**.006**	**-4.445**	**.000**
c15	-.025	.044	-.569	.569
c	-.054	.321	-.168	.867
c'	-.145	.327	-.443	.658
Indirect Effects (with Bootstrap 95% Confidence Interval and Standard Errors)				
	Effect	LL 95%CI	UL 95% CI	SE
Facebook → M → Y	.004	-.001	.010	.003
Snapchat → M → Y	.003	-.001	.008	.002
**Instagram → M → Y**	**.003**	**.000**	**.006**	**.001**
TikTok → M → Y	.002	-.002	.007	.002
LinkedIn → M → Y	-.003	-.101	.120	.054
Twitter → M → Y	.004	-.001	.012	.003
YouTube → M → Y	-.003	-.007	.001	.002
Pinterest → M → Y	.067	-.008	.201	.050
Reddit → M → Y	-.005	-.023	.023	.011
Twitch → M → Y	.006	-.006	.028	.009
Discord → M → Y	-.002	-.041	.037	.019
Dating Apps → M → Y	.018	-.025	.089	.029
Other Social Apps→ M → Y	-.003	-.020	.013	.008

WhatsApp → M → Y	-.002	-.006	.001	.001
iOS Messages → M → Y	.002	-.012	.020	.008

Note— 5,000 bootstraps. Bolded paths are significant.

US lockdown enforcement

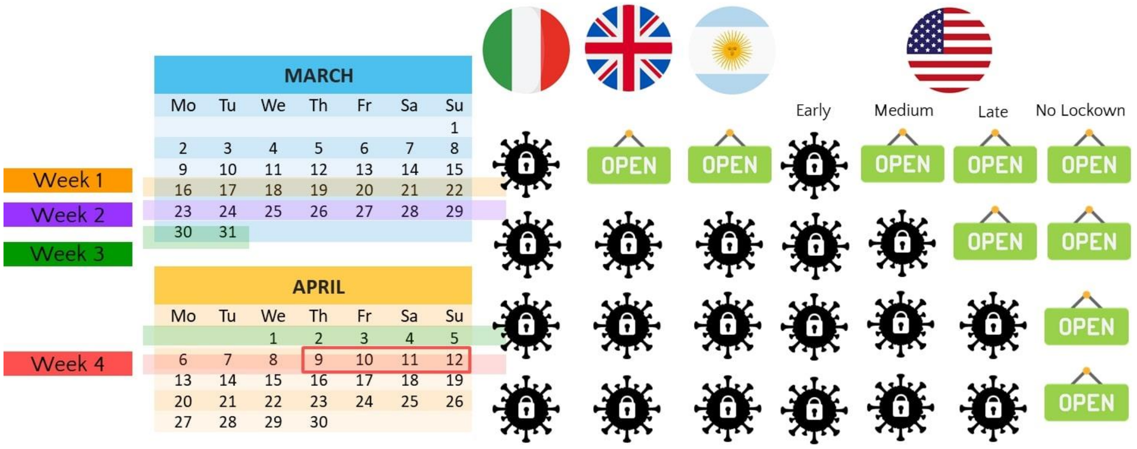

Secondary Data on Mobility

Secondary data on mobility (https://www.google.com/covid19/mobility/) suggests that when we collected our data (March – April 2020), all our participants from Argentina, Italy and UK, were under stringent lockdown protocols and did comply with such protocols. As such we believe that the first lockdown wave was the perfect setting for us to study the effect of lockdown enforcement on social network use and its consequences. Compliance clearly declined later in the year, but also lockdown measures were less stringent and many activities remained available for people to gather safely.

How did the number of visitors change since the beginning of the pandemic?, United Kingdom

Our World in Data

This data shows how community movement in specific locations has changed relative to the period before the pandemic.

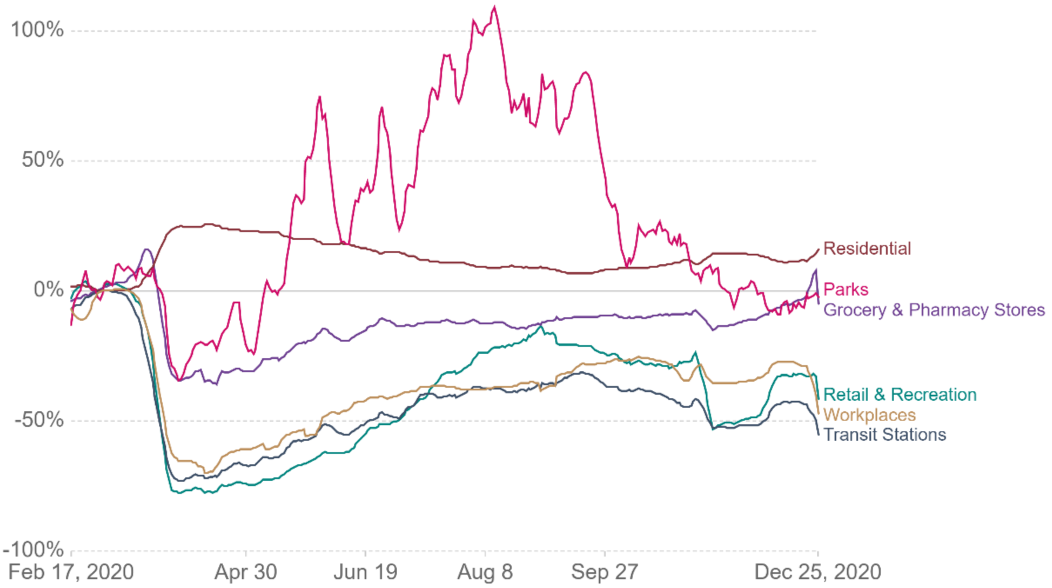

Source: Google COVID-19 Community Mobility Trends – Last updated 28 December, 19:02 (London time)
Note: It's not recommended to compare levels across countries; local differences in categories could be misleading.
OurWorldInData.org/coronavirus • CC BY

How did the number of visitors change since the beginning of the pandemic?, Italy

Our World in Data

This data shows how community movement in specific locations has changed relative to the period before the pandemic.

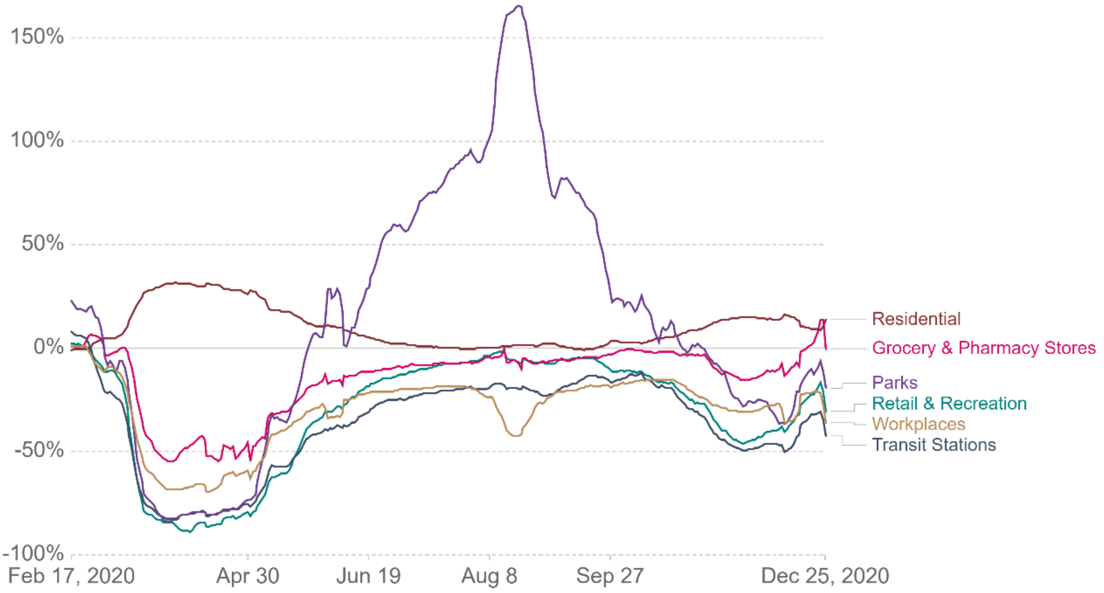

Source: Google COVID-19 Community Mobility Trends – Last updated 28 December, 19:02 (London time)
Note: It's not recommended to compare levels across countries; local differences in categories could be misleading.
OurWorldInData.org/coronavirus • CC BY

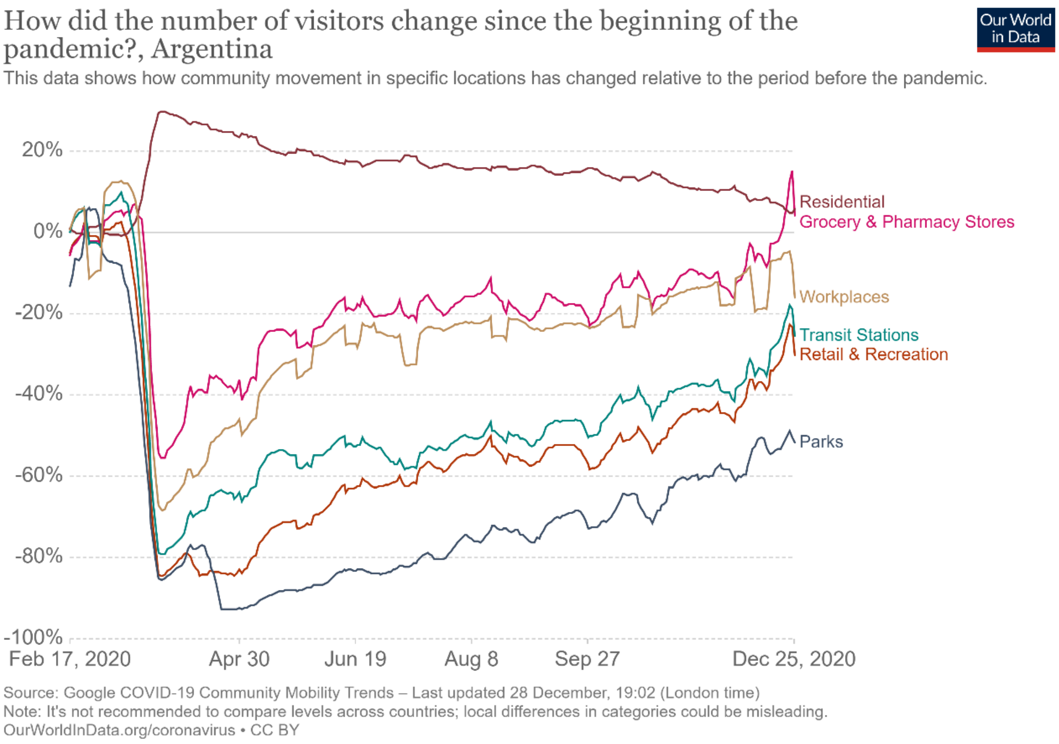

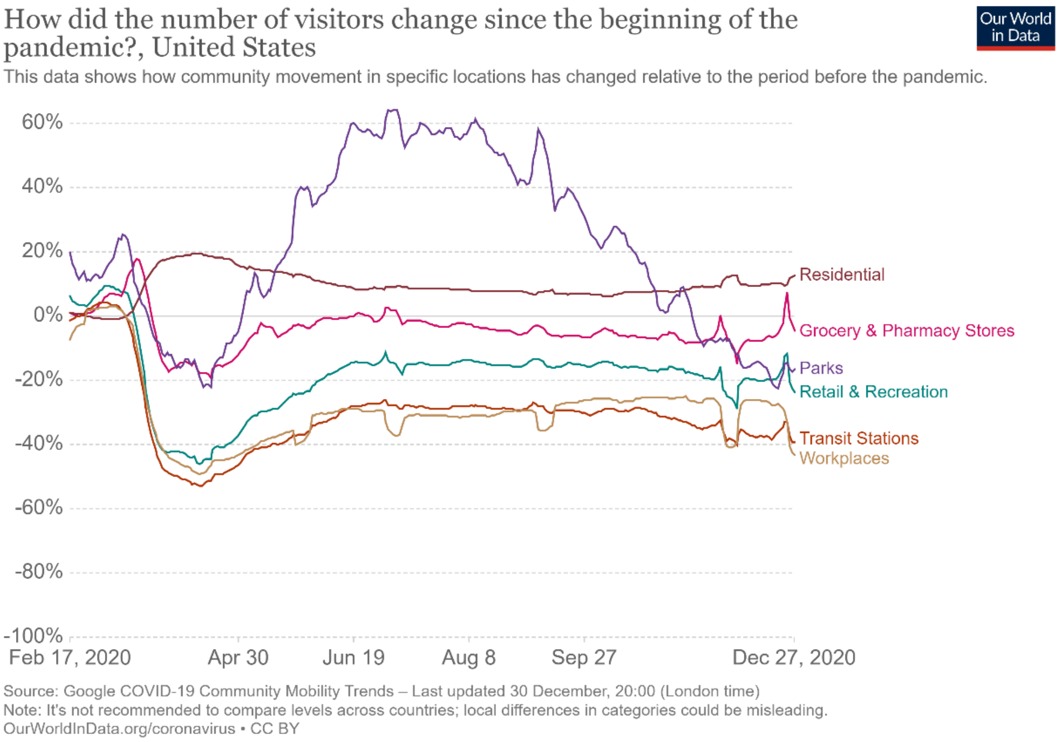

Sources:

https://www.google.com/covid19/mobility/

https://ourworldindata.org/covid-mobility-trends
